# Impact of obesity on patients undergoing surgery for rectal cancer in Australia and New Zealand

**DOI:** 10.1007/s00384-023-04447-0

**Published:** 2023-06-08

**Authors:** Phillip F Yang, Zhen Hao Ang, Sarit Badiani, Christophe R Berney, Matthew J Morgan

**Affiliations:** 1 South Western Sydney Clinical School, Faculty of Medicine, University of New South Wales, Sydney, Australia; 2 Department of Surgery, Bankstown-Lidcombe Hospital, Sydney, Australia

**Keywords:** Rectal cancer, Obesity, Colon, rectum, Proctectomy, Postoperative complications

## Abstract

**Purpose:**

Patients with obesity undergoing rectal cancer surgery may have an increased risk of developing complications, though evidence is inconclusive. The aim of this study was to determine the direct impact of obesity on postoperative outcomes using data from a large clinical registry.

**Method:**

The Binational Colorectal Cancer Audit registry was used to identify patients who underwent rectal cancer surgery in Australia and New Zealand from 2007–2021. Primary outcomes were inpatient surgical and medical complications. Logistic regression models were developed to describe the association between body-mass index (BMI) and outcomes.

**Results:**

Among 3,708 patients (median age 66 years [IQR 56.75–75], 65.0% male), 2.0% had a BMI < 18.5 kg/m^2^, 35.4% had a BMI of 18.5–24.9 kg/m^2^, 37.6% had a BMI of 25.0–29.9 kg/m^2^, 16.7% had a BMI of 30.0–34.9 kg/m^2^, and 8.2% had a BMI ≥ 35.0 kg/m^2^. Surgical complications occurred in 27.7% of patients with a BMI of 18.5–24.9 kg/m^2^, 26.6% of patients with a BMI of 25.0–29.9 kg/m^2^ (OR 0.91, 95% CI 0.76–1.10), 28.5% with a BMI of 30.0–34.9 kg/m^2^ (OR 0.96, 95% CI 0.76–1.21), and 33.2% with a BMI ≥ 35.0 kg/m^2^ (OR 1.27, 95% CI 0.94–1.71). Modelling BMI as a continuous variable confirmed a J-shaped relationship. The association between BMI and medical complications was more linear.

**Conclusion:**

Risk of postoperative complications is increased in patients with obesity undergoing rectal cancer surgery.

**Supplementary Information:**

The online version contains supplementary material available at 10.1007/s00384-023-04447-0.

Obesity is an increasing problem worldwide, including in Australia and New Zealand. In 2017–18, an estimated 31% of Australians aged 18 and over were considered obese, defined as a body mass index (BMI) of 30 kg/m^2^ and over [1]. This is a marked increase compared to 1995 when just 19% of Australian adults were obese. In New Zealand, an estimated 34.3% of adults were considered obese in 2020–21 [2]. Obesity is also more common in older age groups, with a prevalence of 41% in Australian adults aged 65–74 having obesity compared with 16% of adults aged 18–24 [1].

In rectal cancer surgery, patients who have obesity may present technical challenges and are at increased risk of developing postoperative complications. In a recent Australian study by Bell et al. that included 481 patients with rectal cancer, those with obesity were found to have a significantly higher risk of surgical complications (35.6% vs 23.6%) and likelihood of requiring conversion from a minimally invasive surgical approach to open surgery (OR 4.1) [3]. Several studies have shown a similar association between obesity and increased rates of overall complications [4–9] and infective complications [10–12], though others did not [13–19]. Several studies have shown that obesity is a risk factor for conversion to open surgery [13, 19–21], though again others did not [14, 15, 17]. Most studies report no correlation between obesity and histopathological outcomes such as number of lymph nodes harvested or presence of cancer cells at the specimen margins [14–16, 19, 21, 22], or oncological outcomes such as overall survival and disease-free survival [12, 16–18, 21–23]. Unfortunately, the heterogeneous results from this body of evidence, mostly comprising of single-institution cohort studies that differ in geography, methodology, inclusion criteria and definitions of obesity, make it difficult to draw firm conclusions.

More recent studies utilising data from large registries have more consistently demonstrated an association between obesity and adverse outcomes in patients undergoing surgery for rectal cancer. A study by Gebauer et al. of 9,920 patients using prospectively-collected data across 183 German hospitals between 2008 through 2011 showed that obesity was associated with significantly higher risk of overall postoperative (42.9% vs 36.6%) and surgical complications (33.9% vs 26.8%) [24]. Similarly, using data from the Dutch Colorectal Audit that included 20,208 patients across 83 hospitals between 2009 through 2016, Poelemeijer et al. showed that obesity was associated with significantly higher risk of overall complications (43.7% vs 35.1%) and conversion to laparotomy (11.2% vs 4.2%) [25]. Several North American studies using data from the multi-institutional American College of Surgeons National Surgical Quality Improvement Program (ACS-NSQIP) have also reported higher rates of overall complications in patients with obesity [26–29].

The aim of this study was to analyse data from the Binational Colorectal Cancer Audit (BCCA), an Australian and New Zealand clinical registry, looking for variations in outcome in patients undergoing surgery for rectal cancer between those with and without obesity. We hypothesised that patients with obesity are at higher risk of developing postoperative complications.

## Methods

### Study population and study oversight

This study used individual, de-identified data from the BCCA [30]. The BCCA is a prospectively maintained registry of over 43,000 episodes of colorectal cancer patient care and currently captures approximately a quarter of the colorectal cancer operations performed in Australia and New Zealand [31]. Data of patients treated from July 2007 through October 2021 were analysed.

Patients aged 18 years and over who underwent surgery for rectal cancer were included in the study. Rectal cancer, as defined in the registry, was a malignancy that was clinically, radiologically and/or endoscopically considered to be below the rectosigmoid junction, less than or equal to 15 cm from the anal verge. Measurement of body weight and height was not standardised and may have been self-reported in some cases. Patients with a synchronous malignant colorectal neoplasm and those who underwent synchronous resection of another organ at the time of rectal cancer surgery were excluded. After exclusions, 10,502 patients were available for analysis. As the registry’s weight and height fields were not previously mandatory, BMI data was available or able to be calculated for only 3,708 (35.3%) patients, after excluding the top and bottom 0.5% of BMI data to reduce the impact of potential outliers.

This study was approved by the Human Research Ethics Committee of the South Western Sydney Local Health District.

### Outcomes

The primary outcomes of our study were inpatient surgical and medical complications. Definitions of these composite outcomes are provided in Section S1 of the Supplementary Appendix. Secondary outcomes included clinical outcomes (length of hospital stay, unplanned return to theatre, readmission within 30 days) and histopathological outcomes (circumferential and distal margin involvement, distal margin distance, number of lymph nodes harvested).

### Statistical analysis

Our primary analysis evaluated the association of BMI with surgical and medical complications. We constructed two logistic regression models with adjustment for potential confounding factors, including age, sex, American Society of Anesthesiology (ASA) score, height of the cancer from the anal verge, neoadjuvant therapy, T and N stage, operative urgency, seniority of the person performing the surgery, method of surgical entry, and formation of a stoma. The first model treated BMI as a categorical variable according to the standard World Health Organisation (WHO) classification system of weight status. The second model treated BMI as a continuous variable by using a natural cubic spline. Further details including model specifications are provided in Section S2 of the Supplementary Appendix.

In the secondary analyses, we repeated the regression modelling for the outcomes of unplanned return to theatre, readmission within 30 days, and margin involvement. Length of hospital stay, distal margin clearance, and number of lymph nodes harvested were summarised using their medians and interquartile ranges (IQR). All analyses were performed using R software, version 3.6.1 (R Core Team, 2019).

## Results

### Study population

The characteristics of the 3,708 patients, stratified according to BMI cohort, are summarised in Table 1 and their procedures in Supplementary table 1. The median age was 66 years (IQR 56.75–75) and 2,410 (65.0%) patients were male. The median BMI was 26.5 kg/m^2^ (5th to 95th percentile, 19.7–36.8 kg/m^2^), with 925 patients (24.9%) having a BMI ≥ 30.0 kg/m^2^. Half the patients received neoadjuvant therapy. Two-thirds of tumours were located less than 10 cm from the anal verge. Most patients (95.5%) had an elective procedure. The proportions of patients who developed surgical and medical complications were 27.9% and 14.3%, respectively. Inpatient mortality rate was 0.5%.

**Table 1:** Baseline characteristics of the study population

**Characteristic**	**Overall** **(n = 3,708)**	**Body-mass index (kg/m**^**2**^**)**				
		** < 18.5** **(n = 76)**	**18.5–24.9** **(n = 1,312)**	**25.0–29.9** **(n = 1,395)**	**30.0–34.9** **(n = 620)**	** ≥ 35.0** **(n = 305)**
**Age, median (IQR), y**	66 (56.75–75)	64 (54.75–79)	66 (56–77)	66 (57–75)	65 (58–73)	64 (55–71)
**Male sex**	2,410 (65.0)	28 (37)	789 (60.1)	996 (71.4)	431 (69.5)	166 (54.4)
**ASA classification**						
** 1**	538 (14.6)	14 (19)	254 (19.5)	187 (13.5)	66 (10.7)	17 (5.6)
** 2**	1,803 (49.0)	29 (39)	633 (48.6)	715 (51.7)	308 (50.2)	118 (38.8)
** 3**	1,245 (33.9)	28 (38)	383 (29.4)	449 (32.5)	226 (36.8)	159 (52.3)
** 4**	87 (2.4)	3 (4)	31 (2.4)	29 (2.1)	14 (2.3)	10 (3.3)
** 5**	4 (0.1)	0	2 (0.2)	2 (0.1)	0	0
**Tumour location**						
** Low**	792 (21.4)	24 (32)	276 (21.1)	283 (20.3)	141 (22.8)	68 (22.4)
** Middle**	1,620 (43.8)	24 (32)	586 (44.9)	627 (45.1)	271 (43.9)	112 (36.8)
** High**	1,283 (34.7)	28 (37)	444 (34.0)	481 (34.6)	206 (33.3)	124 (40.8)
**Tumour stage**						
** T0**	299 (8.2)	4 (5)	103 (8.0)	111 (8.1)	57 (9.3)	24 (8.0)
** Tis**	22 (0.6)	0	7 (0.5)	15 (1.1)	0	0
** T1**	591 (16.2)	7 (9)	194 (15.0)	226 (16.5)	116 (19.0)	48 (15.9)
** T2**	903 (24.7)	19 (26)	304 (23.5)	346 (25.2)	153 (25.0)	81 (26.9)
** T3**	1,586 (43.4)	35 (47)	571 (44.2)	590 (43.0)	256 (41.8)	134 (44.5)
** T4**	175 (4.8)	3 (4)	76 (5.9)	63 (4.6)	21 (3.4)	12 (4.0)
** TX**	75 (2.1)	6 (8)	37 (2.9)	21 (1.5)	9 (1.5)	2 (0.7)
**Nodal status**						
** N0**	2,327 (64.2)	44 (60)	791 (61.8)	878 (64.4)	419 (68.9)	195 (65.2)
** N1**	846 (23.4)	16 (22)	318 (24.9)	322 (23.6)	130 (21.4)	60 (20.1)
** N2**	317 (8.8)	6 (8)	109 (8.5)	119 (8.7)	50 (8.2)	33 (11.0)
** NX**	132 (3.6)	7 (10)	61 (4.8)	44 (3.2)	9 (1.5)	11 (3.7)
**Distant metastases**						
** M0**	2,836 (77.7)	54 (74)	981 (75.9)	1,078 (78.7)	495 (80.9)	228 (75.7)
** M1**	228 (6.2)	4 (5)	107 (8.3)	76 (5.5)	21 (3.4)	20 (6.6)
** MX**	585 (16.0)	15 (21)	205 (15.9)	216 (15.8)	96 (15.7)	53 (17.6)
**Prognostic stage group**						
** Stage 0**	295 (8.2)	5 (7)	104 (8.1)	118 (8.7)	47 (7.8)	21 (7.0)
** Stage I**	1,208 (33.5)	24 (34)	394 (30.8)	462 (34.1)	220 (36.3)	108 (36.1)
** Stage II**	847 (23.5)	18 (26)	298 (23.3)	308 (22.7)	150 (24.8)	73 (24.4)
** Stage III**	1,034 (28.7)	19 (27)	379 (29.6)	391 (28.9)	168 (27.7)	77 (25.8)
** Stage IV**	225 (6.2)	4 (6)	105 (8.2)	75 (5.5)	21 (3.5)	20 (6.7)
**Neoadjuvant therapy**	1,881 (50.8)	39 (51)	676 (51.6)	699 (50.2)	327 (52.7)	140 (45.9)

Data are presented as no. of patients (%) unless otherwise stated. Percentages may not total 100 because of rounding. Low rectal cancer was defined as < 5 cm from the anal verge, middle 5–9.9 cm and high ≥ 10 cm. Data on preoperative T stage were missing for 18.3% of patients (not shown); on preoperative N stage for 21.8% (not shown); on T stage for 3.6%, N stage for 5.9% and M stage for 17.4%; and on ASA score, tumour location and neoadjuvant therapy for < 1%

*IQR* interquartile range, *ASA* American Society of Anesthesiology

### BMI and risk of surgical and medical complications

**Fig. 1:**
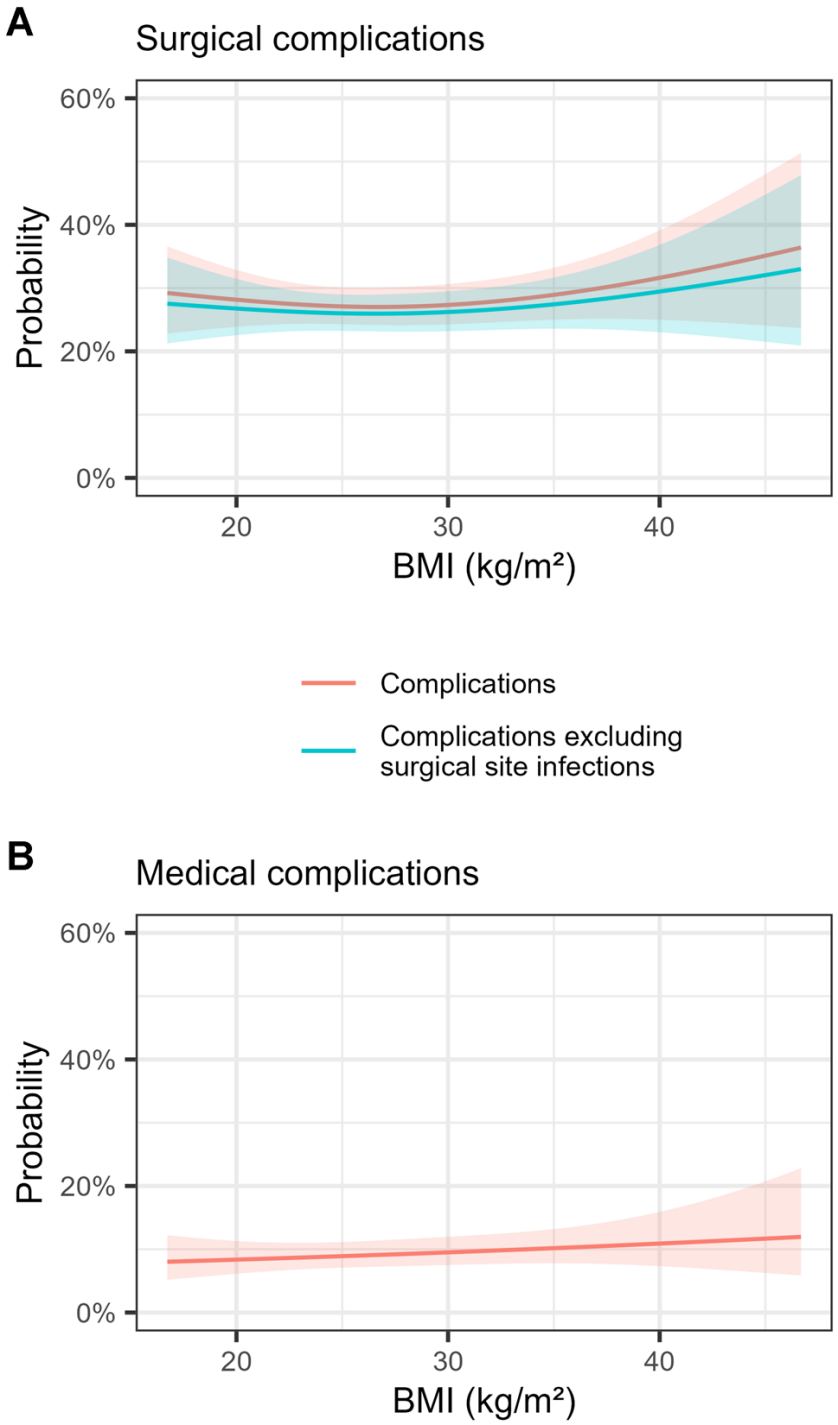
Association between body-mass index (BMI) and post-operative complications. The relationship between BMI and surgical complications (Panel **A**) showed a J-shaped association, whereas that of medical complications (Panel **B**) was more linear. Note that non-focal variables (i.e., confounding factors) are held constant at their mean or reference level, and changes to these parameters will alter the plot. Shaded areas indicate 95% confidence intervals

**Fig. 2:**
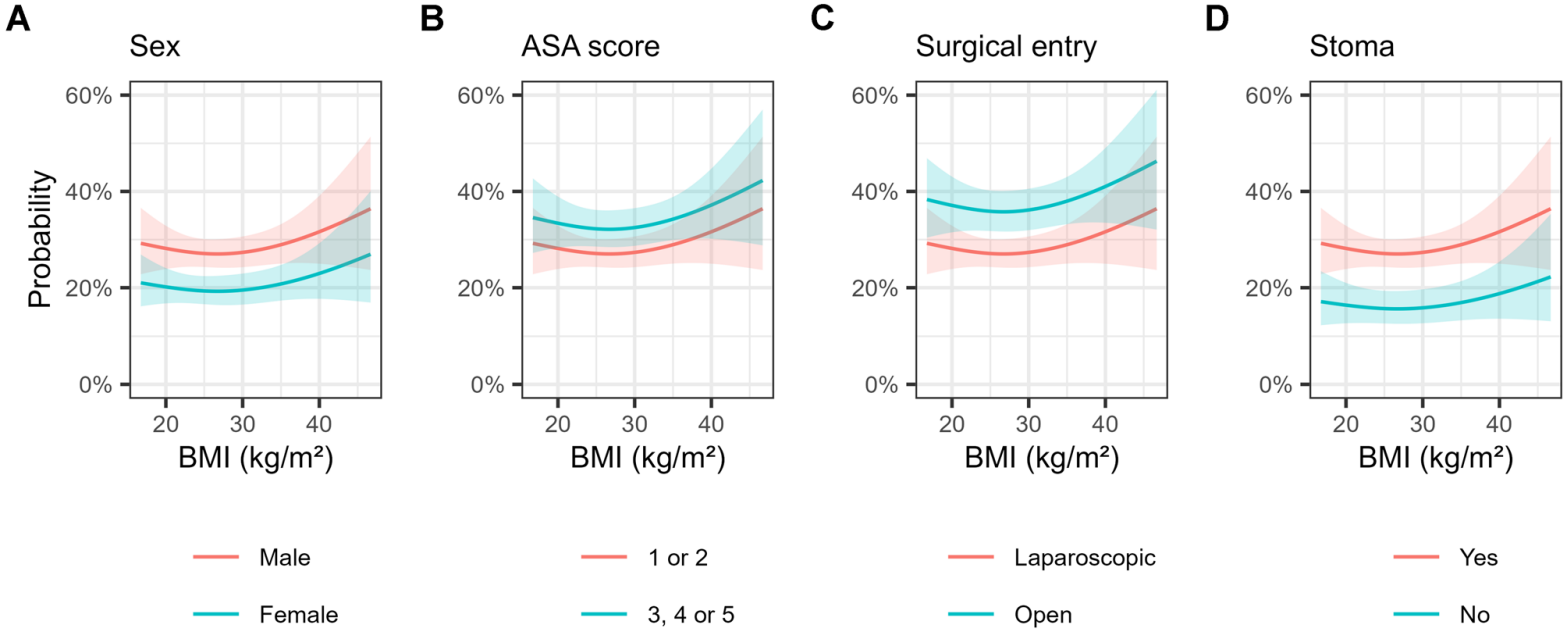
Association between body-mass index (BMI) with surgical complications, stratified according to sex, American Society of Anesthesiology (ASA) score, method of surgical entry, and formation of a stoma. Note that non-focal variables are held constant at their mean or reference level, and changes to these parameters will alter the plot. Shaded areas indicate 95% confidence intervals

Table 2 shows the association between BMI classification and risk of inpatient surgical complications. Surgical complications occurred in 27.7% of patients with a normal BMI of 18.5–24.9 kg/m^2^, 28.5% with a BMI of 30.0–34.9 kg/m^2^ (adjusted OR 0.96, 95% CI 0.76–1.21, *p* = 0.73), and 33.2% with a BMI ≥ 35.0 kg/m^2^ (adjusted OR 1.27, 95% CI 0.94–1.71, *p* = 0.11). Modelling BMI as a continuous variable showed a J-shaped relationship with risk lowest for patients with BMI between 25 and 30 kg/m^2^ (Fig. 1A). Risk of surgical complications was higher in patients who were male (adjusted OR 1.57, 95% CI 1.32–1.87, *p* < 0001), had ASA ≥ 3 (adjusted OR 1.27, 95% CI 1.07–1.51, *p* = 0.006), had an open procedure (adjusted OR 1.50, 95% CI 1.27–1.78, *p* < 0.001), or had proximal diversion (adjusted OR 2.01, 95% CI 1.57–2.61, *p* < 0.001) (Fig. 2). A more linear association was observed between BMI and risk of medical complications (Table 2 and Fig. 1B).

**Table 2:** Association between body-mass index classification and postoperative complications

**Outcome**	**Body-mass index (kg/m**^**2**^**)**				
	** < 18.5**	**18.5–24.9**	**25.0–29.9**	**30.0–34.9**	** ≥ 35.0**
**Surgical complication**	19/73 (26)	338/1,221 (27.7)	350/1,314 (26.6)	167/585 (28.5)	95/286 (33.2)
** Crude OR (95% CI)**	0.92 (0.52–1.55)	Reference	0.95 (0.80–1.13)	1.04 (0.84–1.30)	1.30 (0.98–1.71)
** Adjusted OR (95% CI)**	1.22 (0.67–2.15)	Reference	0.91 (0.76–1.10)	0.96 (0.76–1.21)	1.27 (0.94–1.71)
**Medical complication**	9/73 (12)	165/1,217 (13.6)	187/1,315 (14.2)	96/586 (16.4)	41/287 (14.3)
** Crude OR (95% CI)**	0.90 (0.41–1.75)	Reference	1.06 (0.84–1.33)	1.25 (0.95–1.64)	1.06 (0.73–1.32)
** Adjusted OR (95% CI)**	0.95 (0.40–1.99)	Reference	1.03 (0.82–1.32)	1.22 (0.91–1.63)	0.98 (0.65–1.45)
**Mortality**	1/72 (1)	8/1,226 (0.7)	6/1,320 (0.5)	3/591 (0.5)	0/288

Data are presented as no. of events/no. of patients (%) unless otherwise stated. Data on surgical and medical complications were missing for 6.2% of patients; on mortality for 5.6%

*OR* odds ratio, *CI* confidence interval

Analyses of individual surgical and medical complications are shown in Supplementary table 2 and Supplementary table 3, respectively. The most common surgical complications were prolonged ileus, abdominal/pelvic collections, wound infections, and anastomotic leak, with risk of wound infection being the only one associated with obesity (adjusted OR 1.90, 95% CI 1.07–3.40, *p* = 0.03, for BMI of 30.0–34.9 kg/m^2^; adjusted OR 2.42, 95% CI 1.24–4.64, *p* = 0.008, for BMI ≥ 35 kg/m^2^). After excluding wound infection from the composite outcome, the association of BMI with overall surgical complications still exhibited a J-shaped relationship (Fig. 1A). There was no correlation between BMI classification and length of stay or histopathological outcomes (Table 3). There were J-shaped relationships between BMI and risk of return to theatre and readmission.

**Table 3:** Association between body-mass index classification and clinical and histopathological outcomes

**Outcome**	**Body-mass index (kg/m**^**2**^**)**				
	** < 18.5**	**18.5–24.9**	**25.0–29.9**	**30.0–34.9**	** ≥ 35.0**
**Length of stay, median (IQR), d**	8 (5–12)	8 (6–12)	8 (6–12)	8 (6–12)	8 (6–13)
**Return to theatre**	6/73 (8)	98/1,225 (8.0)	98/1,319 (7.4)	37/591 (6.3)	20/288 (6.9)
** Crude OR (95% CI)**	1.03 (0.39–2.25)	Reference	0.92 (0.69–1.24)	0.77 (0.51–1.13)	0.86 (0.51–1.38)
** Adjusted OR (95% CI)**	1.17 (0.44–2.58)	Reference	0.90 (0.67–1.21)	0.71 (0.47–1.05)	0.87 (0.51–1.41)
**Readmitted within 30 days**	12/72 (17)	156/1,198 (13.0)	160/1,292 (12.4)	77/575 (13.4)	42/284 (14.8)
** Crude OR (95% CI)**	1.34 (0.67–2.45)	Reference	0.94 (0.75–1.20)	1.03 (0.77–1.38)	1.16 (0.79–1.66)
** Adjusted OR (95% CI)**	1.37 (0.69–2.53)	Reference	0.93 (0.73–1.18)	1.02 (0.75–1.36)	1.20 (0.82–1.73)
**Circumferential margin involved**	2/64 (3)	69/1,133 (6.1)	58/1,231 (4.7)	21/547 (3.8)	15/280 (5.4)
** Crude OR (95% CI)**	0.50 (0.08–1.64)	Reference	0.76 (0.53–1.09)	0.62 (0.37–1.00)	0.87 (0.47–1.51)
** Adjusted OR (95% CI)**	0.43 (0.07–1.56)	Reference	0.83 (0.57–1.22)	0.77 (0.44–1.31)	1.12 (0.58–2.07)
**Distal margin involved**	1/70 (1)	12/1,218 (1.0)	13/1,309 (1.0)	6/588 (1.0)	1/291 (0.3)
** Crude OR (95% CI)**	1.46 (0.08–7.56)	Reference	1.01 (0.46–2.25)	1.04 (0.36–2.68)	0.35 (0.02–1.77)
** Adjusted OR (95% CI)**	1.12 (0.06–7.00)	Reference	1.34 (0.57–3.23)	1.72 (0.56–4.89)	0.42 (0.02–2.35)
**Distal margin distance, median (IQR), mm**	30 (18–40)	24 (13–40)	23.5 (12–35)	25 (12–40)	25 (14–40)
**Number of lymph nodes harvested, median (IQR)**	17 (12.25–19.75)	15 (11.75–21)	15 (12–20)	16 (12–21)	16 (12–22)

Data are presented as no. of events/no. of patients (%) unless otherwise stated. Data on length of stay were missing for 6.4% of patients; on return to theatre for 5.7% of patients; on readmissions for 7.7%; on circumferential margin involvement for 12.2%; on distal margin involvement for 6.3%; on distal margin distance for 28.2%; and on number of lymph nodes harvested for 5.8%. IQR, interquartile range; OR, odds ratio; CI, confidence interval

### Patients with missing BMI data

Of the 10,502 eligible patients in the BCCA registry, 6,756 (64.3%) had missing weight, height and/or BMI data. The characteristics of these patients are summarised in Supplementary table 4. Anthropometric data were more likely to be missing in earlier years, when these were not mandatory fields in the registry. Of patients who had surgery prior to 2012, 91.7% had missing anthropometric data, compared with 56.9% of patients who had surgery from 2012 onwards (OR 8.35, 95% CI 7.17–9.78, *p* < 0.001).

Most baseline characteristics were comparable between patients with and without BMI data, including age, sex, tumour height, T stage, and proportion of patients receiving neoadjuvant therapy (Supplementary table 4). The proportion of patients with ASA ≥ 3 was lower in those with missing BMI data (OR 0.74, 95% CI 0.68–0.80, *p* < 0.001). Surgical complications were slightly greater in patients with missing BMI data (OR 1.20, 95% CI 1.07–1.35, *p* = 0.002). There were no differences in medical complications or any other clinical or oncological outcomes (Supplementary table 5).

## Discussion

In this registry-based study of 3,708 patients who underwent rectal cancer surgery, we used exploratory modelling to demonstrate a J-shaped association between BMI and risk of surgical complications. This association was present across subgroups defined by age, ASA score, use of minimally invasive surgery and proximal diversion, which were themselves also associated with higher risk of complications. There was a similar association between BMI and risk of return to theatre and readmission. We found no correlation between BMI and length of stay and histopathological outcomes.

Our study replicated the findings of previous studies but, uniquely, modelled BMI as a continuous variable and thereby demonstrated the presence of an obesity paradox. Almost all previously published registry-based studies [24–27, 29] as well as smaller single-institution cohort studies on this topic categorised BMI based on the WHO classification system. One previous study did employ modelling that treated BMI as a continuous variable, however its focus was on the interaction between BMI and the benefits of laparoscopy, rather than the association of BMI with complications [28]. In general, categorisation of continuous variables is problematic because it involves multiple hypothesis testing with pairwise comparisons of groups, leading to an increased chance of a false positive result, and it also produces an unrealistic step function of risk while assuming homogeneity of risk within groups [32]. In our study, we used BMI categories to perform a preliminary assessment of the relationship between BMI and outcomes, but we also modelled BMI as a continuous variable and demonstrated that the relationship between BMI and risk of surgical complications was in the shape of a J-curve. This so-called obesity paradox, where lower rates of complications are seen in overweight patients compared with underweight or health-weight patients, is a well-recognised phenomenon and has been described in previous studies in the context of rectal cancer surgery [26], but it has never been modelled as clearly as in our study. Finally, some previous studies suggested that increased complications associated with obesity were primarily driven by increased risk of surgical site infections [24, 29], however we showed that the J-shaped association of BMI with surgical complications was still preserved even after excluding wound infections.

Several mechanisms have been proposed to explain higher rates of surgical complications seen in patients with obesity, especially in relation to surgical site infections. Increased subcutaneous adiposity in patients with obesity predisposes them to wound infections because of comparatively lower oxygen perfusion and concentration [33]. Obesity is also associated with impaired immunity, with decreased lymphocyte function and suboptimal neutrophil oxidative killing ability being implicated, which may result in increased infective complications following surgery [34, 35]. Increasing degrees of obesity have also been associated with lower therapeutic tissue concentrations of prophylactic antibiotics [36]. Indirectly, obesity is also associated with related comorbidities such as insulin resistance and diabetes, which are risk factors for postoperative infections [35]. Unfortunately, information on antibiotic dosing and other risk factors for surgical site infections are not captured in the BCCA registry and so could not be factored into our analysis.

The methodology and results of our study differ in several ways from those of previous large registry-based studies (Table 4). The BCCA registry analysed in our study is most similar to the German and Dutch registries analysed by Gebauer et al. [24] and Poelemeijer et al. [25], respectively. All these registries contain data on tumour characteristics and surgical complications, with data collected by clinical staff. The crude incidence of surgical complications was lower in Poelemeijer et al. than in both our study and in Gebauer et al. This is likely due to differences in the definition of the primary outcome. In contrast, the primary outcome of overall complications that was used in the three North American studies using ACS-NSQIP datasets [26, 27, 29] is not directly comparable with the primary outcome of our study. Nonetheless, a key strength of the ACS-NSQIP is that participating hospitals must employ specially trained and independent data collectors, which increases the reliability of its data. This likely explains why rates of surgical site infections reported in the ACS-NSQIP studies [27, 29] are considerably higher than those reported in Gebauer et al. [24] and in our study. Unfortunately, each of the three ACS-NSQIP studies used different procedural codes for their inclusion criteria which further limits direct comparisons. This is most apparent in the higher proportion of patients with low rectal cancer and who underwent abdominoperineal resection in the ACS-NSQIP studies compared with our study. Nonetheless, despite these differences and the previously discussed drawbacks of treating BMI as a categorical variable, all these previous studies demonstrated an association between obesity and overall risk of complications.

**Table 4:** Comparison of registry-based studies of the impact of obesity on outcomes of patients undergoing surgery for rectal cancer

**Study**	**N**	**APR (%)**	**Body-mass index**		**Overall complications**		**Surgical complications**		**Margin involvement**	
			**Category (kg/m**^**2**^**)**	**%**	**Incidence (%)**	**Adjusted OR (95% CI)**	**Incidence (%)**	**Adjusted OR (95% CI)**	**Circumferential (%)**	**Distal (%)**
**Gebauer et al.** [24]	9,920		< 18.5	2.1	43.4	1.16 (0.75–1.80)	28.3	—	5.9	3.4
			18.5–24.9	38.0	36.6	Ref	26.8	—	4.2	0.9
			25.0–29.9	40.5	36.8	1.04 (0.92–1.19)	28.7	—	3.1	0.5
			≥ 30.0	19.4	42.9	1.31 (1.12–1.55)	33.9	—	2.7	0.9
**Hrabe et al.** [27]	5,570*	32.4	< 18.5	4.2	43.7	0.90 (0.66–1.20)				
			18.5–24.9	35.9	38.1	Ref				
			25.0–29.9	32.7	37.3	1.03 (0.90–1.19)				
			30.0–34.9	17.0	44.0	1.36 (1.14–1.62)				
			35.0–39.9	6.5	50.8	1.99 (1.54–2.54)				
			≥ 40.0	3.7	46.6	1.42 (1.02–1.96)				
**Poelemeijer et al.** [25]	20,208		18.5–24.9	40.5	35.1	—	17.3	—		
			25.0–29.9	41.5	37.7	—	18.0	—		
			≥ 30.0	16.4	43.7	—	20.1	—		
**Smith et al.** [26]	11,995	26.9	< 20.0	10.5	35.1	0.99 (0.86–1.15)				
			20.0–24.9	32.0	32.2	Ref				
			25.0–29.9	31.8	—	1.06 (0.96–1.18)				
			30.0–34.9	15.6	37.7	1.30 (1.15–1.47)				
			≥ 35.0	9.8	44.8	1.63 (1.41–1.89)				
**Sweigert et al.** [29]	2,241	52.5	18.5–24.9	33.4	39.2	Ref			8.8	1.9
			25.0–29.9	33.5	42.9	—			6.8	2.5
			30.0–34.9	21.1	39.6	0.87 (0.67–1.12)			6.0	1.5
			≥ 35.0	12.0	53.0	1.44 (1.05–1.96)			4.1	1.5

Data (including odds ratios) not available in some instances

*APR* abdominoperineal resection, *OR* odds ratio, *CI* confidence interval

*Includes patients undergoing surgery for both benign conditions and cancer

Our study has several limitations. First, 64.3% of potentially eligible patients were excluded due to missing BMI data. Our analysis of patients with missing BMI data showed that neither their baseline characteristics nor tumour characteristics differed greatly from those of the analysed cohort. Missingness of BMI data was most correlated with year, with nearly ubiquitous missing BMI data in the early years of the BCCA registry. Whether the excluded patients differed substantively from the analysed cohort and resulted in selection bias is unknown, however we feel that there are no strong signals from the observable data to suggest this to be the case. We have no reason to believe the BMI data is missing not at random. As a result, the main effect of the missing data is likely to be increased standard errors and reduced precision of the estimates, rather than a substantial alteration to the relationships we identified between BMI and complications. Second, the reliance on clinical staff for data collection might have introduced measurement bias. Notably, rates of surgical site infections are significantly lower than reported in comparable studies [3, 27, 29]. Third, we could not adjust for potentially important risk factors such as smoking, long-term steroid use and other comorbidities because these are not captured in the registry. Similarly, information on use of enhanced recovery after surgery protocols, which may reduce postoperative complications, are not captured and so could not be factored into our analysis. Fourth, data on ethnic and socioeconomic background are not captured in the registry, which may limit the transferability of our results. Fifth, the small number of patients with BMI less than 18.5 kg/m^2^ and greater than 40.0 kg/m^2^ resulted in wide confidence intervals for the estimates of effect size at the extremes of BMI. Sixth, though less than 7% of data was missing for the primary composite outcomes, there was a large proportion of missing data on individual surgical and medical complications. Coupled with the low event counts for some of these complications, we therefore could not meaningfully analyse or draw conclusions on these. Finally, we caution that all our models were exploratory rather than predictive, whereby the goal was to describe the association between BMI and outcome rather than address questions of prognosis. Any changes to the values of the non-focal variables of the models depicted in Figs. 1 and 2 will change the outcome, and so these plots should not be used for prognostic purposes.

Our study shows that BMI is an important modifiable risk factor for complications in patients undergoing surgery for rectal cancer. Given that half of the patients in our study received neoadjuvant therapy, this would be an ideal time for interventions that help patients with obesity achieve a healthier weight. Evidence on prehabilitation in rectal cancer surgery is limited, but intervening during neoadjuvant treatment appears feasible [37]. A specific example is the ADIPOSe trial, an Australian multicentre RCT that compared using a very low energy high protein diet versus usual care prior to surgery for rectal cancer in patients with obesity. Unfortunately, this study failed to reach its recruitment target [38]. Based on the results of our modelling, future trials in this area should select a higher BMI threshold (e.g., 35 kg/m^2^ or higher) for their inclusion criteria, as patients with BMI 30.0–34.9 kg/m^2^ had a similar rate of complications as patients with normal BMI.

## Conclusion

Our study showed that patients with obesity were at higher risk of developing postoperative complications following surgery for rectal cancer. Patients who were underweight were also at greater risk. These findings may support the importance of prehabilitation programs for high-risk patients. The main limitation of the study is the large amount of missing data within the BCCA registry. Further research is required to verify our findings using more complete datasets.

### Supplementary Information

Below is the link to the electronic supplementary material.

Supplementary file1 (PDF 183 KB)
