# Supplementary material for: Impact of obesity on patients undergoing surgery for rectal cancer in Australia and New Zealand
Source: Int J Colorectal Dis. 2023 Jun 8;38(1):163. doi: 10.1007/s00384-023-04447-0 (PMC10250449; doi:10.1007/s00384-023-04447-0)
Supplement: Supplementary file 1 — Supplementary file1 (PDF 183 KB) [file 384_2023_4447_MOESM1_ESM.pdf]

Supplementary Appendix

Section S1. Definitions of outcomes

Inpatient surgical complication was a composite outcome comprising abdominal or pelvic collection greater than 3 cm in diameter seen on computed tomography or ultrasound; anastomotic leak, based on clinical or radiological evidence; enterocutaneous fistula; superficial wound dehiscence; deep wound dehiscence; surgical site infection, defined as superficial or deep wound infection where pus is released from the wound or the presence of wound cellulitis and temperature greater than 38°C; sepsis; prolonged ileus, defined as a delay in bowel function of greater than one week following surgery; mechanical small bowel obstruction based on radiological evidence or proven at surgery; urinary retention requiring re-catheterisation; ureteric injury; splenectomy; post-operative haemorrhage requiring intervention, including transfusion, return to theatre, drainage or radiological intervention; or any other significant surgical complication.

Inpatient medical complication was a composite outcome comprising deep vein thrombosis and/or pulmonary embolism; chest infection requiring antibiotics and any two of the following clinical features: (a) chest x-ray changes, (b) bronchial breathing, or (c) temperature greater than 38°C; any of the following cardiac complications: (a) arrhythmia, (b) acute pulmonary oedema, (c) myocardial infarction, or (d) cardiac arrest; or any other significant medical complication.

Section S2. Detailed statistical methods

Dependent variables

All suitable dependent variables that were available in the BCCA dataset were used: BMI, age, sex, ASA score, height of rectal cancer in centimetres from the anal verge, neoadjuvant therapy, T stage, N stage, operative urgency (elective or non-elective), most senior person performing the surgery (consultant surgeon, fellow or registrar), method of surgical entry (minimally-invasive approach or laparotomy), and formation of a stoma.

Model development

We developed a sequential series of logistic regression models, treating BMI separately a categorical and continuous variable. We used a combination of clinical guidance and hybrid stepwise selection to determine variables to include in the model. Hybrid stepwise selection starts with a multivariable model containing all candidate variables. Variables are then removed one at a time as for backward selection, but at each step, any variables removed in previous steps are added back in and checked for model fit improvement based on the Akaike information criterion. This process is repeated until model fit is no longer improved by the addition or removal of any further variables. This produced a preliminary main effects model for surgical complications containing sex, ASA score, tumour height, N stage, method of surgical entry, and formation of a stoma as covariates; and a model for medical complications containing sex, ASA score, N stage, method of surgical entry, formation of a stoma, and use of neoadjuvant therapy as covariates. Missing data were handled using complete case analysis.

The preliminary main effects models were then modified in the following ways:

- BMI and age were re-introduced because these were considered important variables
- BMI was examined separately as a categorical and continuous variable. As a categorical variable, the World Health Organisation classification system of weight status was used: underweight (< 18.5 kg/m²), normal weight (18.5–24.9 kg/m²), overweight (25.0–29.9 kg/m²), obesity class I (30.0–34.9 kg/m²), and obesity class II or greater (≥ 35 kg/m²). Obesity class II (35.0–39.9 kg/m²) and obesity class III (≥ 40 kg/m²) were grouped together because of the small number of cases in the latter. As a continuous variable, BMI was fitted with a natural cubic spline with one knot at the median. Using a natural cubic spline performed better than a fractional polynomial term and using one knot at the median performed better than functions with a larger number of knots.
- ASA score was dichotomised to < 3 and ≥ 3 because the coefficient of the original variable (with five levels) was not significant
- N stage was dichotomised to N0 and N1/N2 because the coefficient of the original variable (with three levels) was not significant
- Interaction terms between BMI and the other covariates were examined but these did not meaningfully improve model performance or interpretation

Thus, the final model for surgical complications contained BMI, age, sex, ASA score, tumour height, N stage, method of surgical entry, and formation of a stoma as covariates; and the model for medical complications contained BMI, age, sex, ASA score, N stage, method of surgical entry, formation of a stoma, and use of neoadjuvant therapy as covariates.

Interpretation of results

The purpose of constructing the regression models was exploratory rather than predictive, that is, we were interested in investigating the association between BMI and outcome rather than address questions of prognosis. This was why no attempt was made to validate the models. However, because the regression coefficients of non-linear terms cannot be easily summarised (e.g., by using odds ratios), the best way to convey the effect of BMI on outcome was to visualise the model using effect plots. Hence, the marginal effect of BMI was plotted after holding the non-focal variables (i.e., confounding factors) constant and varies only the focal variable (i.e., BMI). Here, we held the non-focal continuous variables constant at their mean and non-focal categorical variables constant at their reference level. Note that the generated predictions differ from the crude complication rates calculated when BMI was treated as a categorical variable. This is because the non-focal variables held constant at their reference levels presents the most favourable of tumour-, surgeon- and surgery-related conditions from a clinical perspective, hence generating lower than average complication rates. Choosing any other reference level would alter the magnitude but not the overall shape of the plots.

Secondary analyses

The secondary analyses evaluated the association of BMI with clinical and histopathological outcomes. Binary categorical outcomes (unplanned return to theatre, readmission within 30 days, circumferential and distal margin involvement) were analysed using logistic regression models, with BMI again treated separately as a categorial and continuous variable, and using the same method as that used for studying the primary outcomes, as described above. Continuous outcomes (length of hospital stay, distal margin distance, number of lymph

nodes harvested) were not normally distributed and so were summarised using their medians and interquartile ranges.

Section S3. Supplementary tables

Supplementary table 1. Surgical details.

Characteristic	Overall (n = 3,708)	Body mass index (kg/m²) < 18.5 (n = 76)	18.5–24.9 (n = 1,312)	25.0–29.9 (n = 1,395)	30.0–34.9 (n = 620)	≥ 35.0 (n = 305)
**Operative urgency**						
**Emergency**	56 (1.5)	0	28 (2.1)	19 (1.4)	6 (1.0)	3 (1.0)
**Urgent**	111 (3.0)	9 (12)	42 (3.2)	41 (2.9)	13 (2.1)	6 (2.0)
**Elective**	3,536 (95.5)	67 (88)	1,239 (94.7)	1,333 (95.7)	601 (96.9)	296 (97.0)
**Most senior person performing operation**						
**Consultant**	2,827 (76.7)	55 (72)	977 (74.8)	1,081 (78.0)	478 (78.0)	236 (77.9)
**Fellow**	823 (22.3)	21 (28)	318 (24.3)	293 (21.1)	128 (20.9)	63 (20.8)
**Registrar**	35 (0.9)	0	12 (0.9)	12 (0.9)	7 (1.1)	4 (1.3)
**Surgical entry**						
**Open**	829 (22.5)	16 (21)	287 (22.0)	272 (19.6)	148 (24.2)	106 (35.2)
**Laparoscopic**	1,757 (47.7)	51 (67)	693 (53.1)	652 (47.0)	252 (41.2)	109 (36.2)
**Hybrid**	523 (14.2)	5 (7)	161 (12.3)	233 (16.8)	93 (15.2)	31 (10.3)
**Conversion of laparoscopic**	194 (5.3)	1 (1)	38 (2.9)	80 (5.8)	52 (8.5)	23 (7.6)
**Robotic**	127 (3.5)	1 (1)	45 (3.4)	48 (3.5)	22 (3.6)	11 (3.7)
**Transanal**	143 (3.9)	2 (3)	45 (3.4)	64 (4.6)	20 (3.3)	12 (4.0)
**taTME**	108 (2.9)	0	36 (2.8)	38 (2.7)	25 (4.1)	9 (3.0)
**Procedure**						
**Proctocolectomy**	28 (0.8)	1 (1)	12 (0.9)	7 (0.5)	6 (1.0)	2 (0.7)
**High anterior resection**	157 (4.3)	6 (8)	58 (4.5)	66 (4.8)	15 (2.4)	12 (4.0)
**Low anterior resection**	684 (18.6)	12 (16)	234 (18.1)	256 (18.4)	116 (18.9)	66 (21.8)
**Ultra-low anterior resection**	1,758 (47.8)	27 (36)	624 (48.2)	679 (48.9)	305 (49.7)	123 (40.6)
**APR**	623 (17.0)	16 (21)	201 (15.5)	228 (16.4)	113 (18.4)	65 (21.5)
**Hartmann's**	90 (2.4)	3 (4)	26 (2.0)	30 (2.2)	20 (3.3)	11 (3.6)

Characteristic	Overall (n = 3,708)	Body mass index (kg/m²)				
		< 18.5 (n = 76)	18.5–24.9 (n = 1,312)	25.0–29.9 (n = 1,395)	30.0–34.9 (n = 620)	≥ 35.0 (n = 305)
Colo-anal anastomosis	64 (1.7)	0	26 (2.0)	26 (1.9)	10 (1.6)	2 (0.7)
Local excision	30 (0.8)	1 (1)	14 (1.1)	9 (0.6)	5 (0.8)	1 (0.3)
TEMS/TAMIS	114 (3.1)	1 (1)	35 (2.7)	55 (4.0)	13 (2.1)	10 (3.3)
Other	126 (3.4)	8 (11)	64 (4.9)	32 (2.3)	11 (1.8)	11 (3.6)
**Stoma formation**						
No	764 (20.9)	14 (19)	277 (21.5)	297 (21.6)	105 (17.3)	71 (23.5)
Yes	2,821 (77.2)	57 (76)	987 (76.5)	1,055 (76.6)	495 (81.4)	227 (75.2)
Already present	68 (1.9)	4 (5)	26 (2.0)	26 (1.9)	8 (1.3)	4 (1.3)

Data are presented as no. (% within group) unless otherwise stated. Percentages may not total 100 because of rounding. Data on operative urgency, seniority of the person performing the surgery, method of surgical entry and procedure were missing for < 1% of patients; on stoma formation for 1.4%. taTME, transanal total mesorectal excision; APR, abdominoperineal resection; TEMS, transanal endoscopic microsurgery; TAMIS, transanal minimally invasive surgery

Supplementary table 2. Association between body-mass index classification and surgical complications.

Outcome	Body mass index (kg/m²)				
	< 18.5	18.5–24.9	25.0–29.9	30.0–34.9	≥ 35.0
**Surgical complication**	19/73 (26)	338/1,221 (27.7)	350/1,314 (26.6)	167/585 (28.5)	95/286 (33.2)
**Crude OR (95% CI)**	0.92 (0.52–1.55)	Reference	0.95 (0.80–1.13)	1.04 (0.84–1.30)	1.30 (0.98–1.71)
**Adjusted OR (95% CI)**	1.22 (0.67–2.15)	Reference	0.91 (0.76–1.10)	0.96 (0.76–1.21)	1.27 (0.94–1.71)
**Abdominal/pelvic collections**	2/26 (8)	54/451 (12)	60/488 (12.3)	26/230 (11.3)	15/100 (15)
**Crude OR (95% CI)**	0.61 (0.10–2.14)	Reference	1.03 (0.70–1.53)	0.94 (0.56–1.53)	1.02 (0.54–1.83)
**Adjusted OR (95% CI)**	0.78 (0.12–2.81)	Reference	1.07 (0.70–1.63)	0.97 (0.56–1.63)	1.01 (0.51–1.88)
**Anastomotic leak**	1/22 (4)	39/365 (10.7)	33/391 (8.4)	20/182 (11.0)	14/94 (15)
**Crude OR (95% CI)**	0.40 (0.02–1.99)	Reference	0.77 (0.47–1.25)	1.03 (0.57–1.81)	1.46 (0.74–2.77)
**Adjusted OR (95% CI)**	0.55 (0.03–2.86)	Reference	0.71 (0.43–1.18)	0.85 (0.45–1.55)	1.27 (0.62–2.51)
**Enterocutaneous fistula**	0/26	2/450 (0.4)	1/487 (0.2)	0/230	0/123
**Superficial wound dehiscence**	1/26 (4)	24/450 (5.3)	37/487 (7.6)	17/230 (7.4)	8/123 (6.5)
**Crude OR (95% CI)**	0.71 (0.04–3.58)	Reference	1.46 (0.86–2.51)	1.42 (0.73–2.68)	1.23 (0.51–2.71)
**Adjusted OR (95% CI)**	0.87 (0.05–4.93)	Reference	1.71 (0.95–3.16)	1.39 (0.68–2.84)	0.89 (0.35–2.13)
**Deep wound dehiscence**	1/26 (4)	9/450 (2.0)	6/487 (1.2)	6/230 (2.6)	3/123 (2)
**Crude OR (95% CI)**	1.96 (0.10–11.05)	Reference	0.61 (0.20–1.71)	1.31 (0.44–3.69)	1.23 (0.27–4.18)
**Adjusted OR (95% CI)**	1.25 (0.06–8.26)	Reference	0.51 (0.17–1.46)	0.80 (0.24–2.42)	0.78 (0.61–2.92)
**Wound infection**	1/71 (1)	29/1,208 (2.4)	41/1,306 (3.1)	27/582 (4.6)	19/287 (6.6)
**Crude OR (95% CI)**	0.58 (0.03–2.78)	Reference	1.32 (0.82–2.15)	1.98 (1.15–3.38)	2.88 (1.57–5.18)
**Adjusted OR (95% CI)**	0.86 (0.05–4.32)	Reference	1.45 (0.87–2.48)	1.90 (1.07–3.40)	2.62 (1.35–5.00)
**Sepsis**	2/26 (8)	25/450 (5.6)	24/487 (4.9)	14/230 (6.1)	4/123 (3.3)
**Crude OR (95% CI)**	1.42 (0.22–5.16)	Reference	0.88 (0.49–1.57)	1.10 (0.55–2.13)	0.57 (0.17–1.57)
**Adjusted OR (95% CI)**	2.00 (0.30–7.77)	Reference	0.89 (0.48–1.65)	0.98 (0.45–2.02)	0.61 (0.17–1.69)
**Prolonged ileus**	5/26 (19)	143/452 (31.6)	156/487 (32.0)	65/230 (28.3)	34/123 (27.6)
**Crude OR (95% CI)**	0.51 (0.17–1.29)	Reference	1.02 (0.77–1.34)	0.85 (0.60–1.20)	0.83 (0.53–1.27)
**Adjusted OR (95% CI)**	0.56 (0.18–1.45)	Reference	0.96 (0.72–1.29)	0.86 (0.59–1.25)	0.88 (0.54–1.40)
**Small bowel obstruction**	2/26 (8)	25/450 (5.6)	36/487 (7.4)	8/230 (3.5)	3/123 (2.4)
**Crude OR (95% CI)**	1.42 (0.22–5.16)	Reference	1.36 (0.80–2.32)	0.61 (0.25–1.32)	0.42 (0.10–1.24)
**Adjusted OR (95% CI)**	1.31 (0.20–4.97)	Reference	1.31 (0.76–2.29)	0.44 (0.16–1.05)	0.40 (0.91–1.18)
**Urinary retention**	2/26 (8)	39/450 (8.7)	48/487 (9.9)	22/230 (9.6)	11/123 (8.9)
**Crude OR (95% CI)**	1.34 (0.14–3.11)	Reference	1.15 (0.74–1.80)	1.11 (0.63–1.91)	1.04 (0.49–2.02)
**Adjusted OR (95% CI)**	0.91 (0.14–3.39)	Reference	1.12 (0.71–1.79)	1.08 (0.60–1.89)	1.09 (0.48–2.25)
**Ureteric injury**	0/26	4/450 (0.9)	2/487 (0.4)	2/230 (0.9)	2/123 (1.6)

**Splenectomy**	0/26	1/450 (0.2)	2/487 (0.4)	0/230	1/123 (0.8)
**Postoperative haemorrhage**	1/26 (4)	23/450 (5.1)	13/487 (2.7)	6/230 (2.6)	4/123 (3.3)
**Crude OR (95% CI)**	0.74 (0.04–3.76)	Reference	0.51 (0.25–1.00)	0.50 (0.18–1.17)	0.62 (0.18–1.66)
**Adjusted OR (95% CI)**	—	Reference	0.49 (0.22–1.04)	0.61 (0.21–1.49)	0.59 (0.16–1.68)

Data are presented as no. of events/no. of patients (%) unless otherwise stated. Odds ratios were not computed for models with complete or quasi-complete separation. OR, odds ratio; CI, confidence interval

Supplementary table 3. Association between body-mass index classification and medical complications.

Outcome	Body mass index (kg/m²)				
	**< 18.5**	**18.5–24.9**	**25.0–29.9**	**30.0–34.9**	**≥ 35.0**
**Medical complication**	9/73 (12)	165/1,217 (13.6)	187/1,315 (14.2)	96/586 (16.4)	41/287 (14.3)
**Crude OR (95% CI)**	0.90 (0.41–1.75)	Reference	1.06 (0.84–1.33)	1.25 (0.95–1.64)	1.06 (0.73–1.32)
**Adjusted OR (95% CI)**	0.95 (0.40–1.99)	Reference	1.03 (0.82–1.32)	1.22 (0.91–1.63)	0.98 (0.65–1.45)
**DVT/PE**	0/20	7/281 (2.5)	10/339 (2.9)	5/165 (3.0)	2/70 (2.9)
**Crude OR (95% CI)**	—	Reference	1.19 (0.45–3.32)	1.22 (0.36–3.90)	1.15 (0.17–4.89)
**Adjusted OR (95% CI)**	—	Reference	1.26 (0.44–3.90)	1.30 (0.36–4.54)	0.51 (0.03–3.28)
**Chest infection**	4/20 (20)	38/282 (13.5)	46/339 (13.6)	12/165 (7.3)	7/70 (10)
**Crude OR (95% CI)**	1.61 (0.44–4.66)	Reference	1.01 (0.64–1.61)	0.50 (0.25–0.97)	0.71 (0.28–1.58)
**Adjusted OR (95% CI)**	2.32 (0.60–7.43)	Reference	1.03 (0.63–1.69)	0.45 (0.21–0.92)	0.57 (0.20–1.42)
**Cardiac**	2/20 (10)	48/281 (17.1)	47/339 (13.9)	29/165 (17.6)	13/70 (19)
**Crude OR (95% CI)**	0.54 (0.08–1.95)	Reference	0.78 (0.50–1.21)	1.04 (0.62–1.71)	1.11 (0.54–2.13)
**Adjusted OR (95% CI)**	0.75 (0.11–3.00)	Reference	0.75 (0.46–1.20)	1.09 (0.62–1.90)	1.02 (0.47–2.12)

Data are presented as no. of events/no. of patients (%) unless otherwise stated. Odds ratios were not computed for models with complete separation. OR, odds ratio; CI, confidence interval

Supplementary table 4. Comparison of baseline characteristics of the study population (patients with BMI data) and patients without BMI data.

Characteristic	Patients with BMI data (n = 3,738)	Patients without BMI data (n = 6,756)	Odds ratio (95% CI)
**Age, median (IQR), y**	66 (56–75)	67 (58–75)	
**Male sex**	2,427 (64.8)	4,319 (63.9)	0.96 (0.88–1.04)
**ASA classification**			
**ASA 1 or 2**	2,351 (63.4)	4,515 (70.1)	Reference
**1**	540 (14.6)	1,188 (18.5)	
**2**	1,811 (48.9)	3,327 (51.7)	
**ASA 3, 4 or 5**	1,356 (36.6)	1,923 (29.9)	0.74 (0.68–0.80)
**3**	1,268 (34.0)	1,760 (27.3)	
**4**	90 (2.4)	161 (2.5)	
**5**	4 (0.1)	2 (0.03)	
**Tumour location**			
**Low**	803 (21.6)	924 (21.1)	0.97 (0.88–1.08)
**Middle or high**	2,922 (78.4)	3,452 (78.9)	Reference
**Middle**	1,634 (43.9)	1,905 (43.5)	
**High**	1,288 (34.6)	1,547 (35.4)	
**Tumour stage**			
**T0, Tis, T1 or T2**	1,832 (50.8)	3,041 (49.3)	Reference
**T0**	301 (8.2)	413 (6.5)	
**Tis**	23 (0.6)	83 (1.3)	
**T1**	596 (16.2)	971 (15.2)	
**T2**	912 (24.8)	1,574 (24.6)	
**T3 or T4**	1,772 (49.2)	3,123 (50.7)	1.06 (0.98–1.15)
**T3**	1,595 (43.4)	2,658 (41.5)	
**T4**	177 (4.8)	465 (7.3)	
**TX**	75 (2.0)	83 (1.3)	
**Nodal status**			
**N0**	2,343 (64.2)	3,901 (61.2)	Reference
**N1 or N2**	1,173 (33.4)	2,161 (35.6)	1.11 (1.01–1.21)
**N1**	853 (23.4)	1,519 (23.8)	
**N2**	320 (8.8)	642 (10.1)	
**NX**	134 (3.7)	307 (4.8)	
**Distant metastases**			
**M0**	2,856 (77.7)	4.866 (78.6)	Reference
**M1**	232 (6.3)	568 (9.2)	1.44 (1.23–1.69)
**MX**	589 (16.0)	753 (12.2)	
**Prognostic stage group**			
**Stage 0**	297 (8.2)	437 (7.3)	
**Stage I**	1,222 (33.6)	1,966 (32.8)	
**Stage II**	848 (23.3)	1,323 (22.1)	
**Stage III**	1,042 (28.6)	1,717 (28.6)	
**Stage IV**	229 (6.3)	553 (9.2)	
**Neoadjuvant therapy**	1,897 (50.8)	3,173 (50.3)	0.98 (0.90–1.06)
**Year of surgery, n (% within row)**			
**Pre-2012**	189 (8.3)	2,080 (91.7)	8.35 (7.17–9.78)
**2007**	0	151 (100)	

**2008**	0	411 (100)	
**2009**	2 (0.4)	452 (99.6)	
**2010**	72 (12.1)	525 (87.9)	
**2011**	115 (17.5)	541 (82.5)	
**2012 onwards**	3,549 (43.1)	4,676 (56.9)	Reference
**2012**	173 (27.1)	466 (72.9)	
**2013**	145 (25.8)	418 (74.2)	
**2014**	174 (21.1)	651 (78.9)	
**2015**	233 (27.4)	616 (72.6)	
**2016**	217 (25.5)	633 (74.5)	
**2017**	324 (35.2)	596 (64.8)	
**2018**	583 (56.4)	451 (43.6)	
**2019**	685 (63.3)	398 (36.7)	
**2020**	688 (68.8)	312 (31.2)	
**2021**	327 (70.8)	135 (29.2)	
**Elective operation**	3,565 (95.5)	6,410 (95.3)	0.95 (0.78–1.15)
**Consultant performing operation**	2,850 (76.7)	4,950 (83.6)	1.55 (1.39–1.71)
**Surgical entry**			
**Open**	836 (22.5)	2,995 (45.3)	2.84 (2.59–3.11)
**Other**	2,873 (77.5)	3,623 (54.7)	Reference
**Laparoscopic**	1,771 (47.7)	2,119 (32.0)	
**Hybrid**	524 (14.1)	575 (8.7)	
**Conversion of laparoscopic**	199 (5.4)	257 (3.9)	
**Robotic**	127 (3.4)	161 (2.4)	
**Transanal**	143 (3.9)	388 (5.9)	
**taTME**	109 (2.9)	123 (1.9)	
**Procedure**			
**Proctocolectomy**	28 (0.8)	87 (1.3)	
**High anterior resection**	158 (4.3)	212 (3.2)	
**Low anterior resection**	687 (18.5)	1,232 (18.3)	
**Ultra-low anterior resection**	1,770 (47.8)	2,779 (41.3)	
**APR**	634 (17.1)	1,230 (18.3)	
**Hartmann's**	90 (2.4)	236 (3.5)	
**Colo-anal anastomosis**	64 (1.7)	41 (0.6)	
**Local excision**	30 (0.8)	139 (2.1)	
**TEMS/TAMIS**	114 (3.1)	376 (5.6)	
**Other**	129 (3.5)	356 (5.3)	
**Stoma formation**			
**No**	770 (20.9)	1,316 (20.7)	Reference
**Yes**	2,844 (77.2)	4,969 (78.1)	1.02 (0.92–1.13)
**Already present**	69 (1.9)	76 (1.2)	

Data are presented as no. of patients (% within group) unless otherwise stated. Percentages may not total 100 because of rounding. Low rectal cancer defined as < 5 cm from the anal verge; middle 5–9.9 cm; and high ≥ 10 cm. CI, confidence interval; IQR, interquartile range; taTME, transanal total mesorectal excision; APR, abdominoperineal resection; TEMS, transanal endoscopic microsurgery; TAMIS, transanal minimally invasive surgery.

Supplementary table 5. Comparison of clinical and histopathological outcomes of the study population (patients with BMI data) and patients without BMI data.

Characteristic	Patients with BMI data	Patients without BMI data	Crude OR (95% CI)	Adjusted OR (95% CI)
**Surgical complication**	982/3056 (28.0)	1,713/5,505 (31.1)	1.16 (1.06–1.27)	1.20 (1.07–1.35)
**Medical complication**	505/3,505 (14.4)	732/4,954 (14.8)	1.02 (0.91–1.16)	0.99 (0.85–1.15)
**Length of stay, median (IQR), d**	8 (6–12)	8 (6–13)		
**Return to theatre**	263/3,524 (7.5)	406/6,269 (6.5)	0.86 (0.73–1.01)	0.92 (0.76–1.12)
**Readmitted within 30 days**	455/3,447 (13.2)	510/3,648 (14.0)	1.07 (0.93–1.22)	0.88 (0.75–1.03)
**Circumferential margin involved**	167/3,279 (5.1)	401/5,179 (7.7)	1.56 (1.30–1.89)	1.05 (0.82–1.34)
**Distal margin involved**	34/3.500 (1.0)	104/5,000 (2.1)	2.17 (1.48–3.24)	1.56 (0.93–2.65)
**Distal margin distance, median (IQR), mm**	25 (13–39)	25 (15–40)		
**Number of lymph nodes harvested, median (IQR)**	15 (12–20)	14 (10–20)		

Data are presented as no. of events/no. of patients (%) unless otherwise stated. OR, odds ratio; CI, confidence interval
